# Delayed Microbiological Diagnosis and Fluoroquinolone-Associated Tendon Rupture in Pulmonary Nocardiosis Complicating Autoimmune Pulmonary Alveolar Proteinosis: A Case Report

**DOI:** 10.7759/cureus.104445

**Published:** 2026-02-28

**Authors:** Enrico Fulco

**Affiliations:** 1 Department of Primary Health Care, Internal Medicine Unit Addressed to Frailty and Aging, Ospedale Santa Maria Delle Croci, Ravenna, ITA

**Keywords:** anti-gm-csf antibodies, autoimmune pulmonary alveolar proteinosis, internal medicine, nocardia wallacei, pulmonary nocardiosis

## Abstract

This report describes pulmonary nocardiosis in autoimmune pulmonary alveolar proteinosis (PAP), characterized by delayed microbiological diagnosis and a severe fluoroquinolone-associated adverse reaction. The autoimmune form, mediated by anti-granulocyte-macrophage colony-stimulating factor (GM-CSF) antibodies, predisposes patients to opportunistic infections due to impaired alveolar macrophage function. We describe a 48-year-old male smoker with a history of recurrent respiratory infections who presented with fever and hypoxemic respiratory failure. High-resolution CT demonstrated bilateral ground-glass opacities with superimposed interlobular septal thickening, consistent with a diffuse crazy-paving pattern predominantly involving the lower lobes, associated with focal consolidations in the left lower and right upper lobes. Despite initiation of broad-spectrum antimicrobial therapy, the patient exhibited clinical deterioration characterized by recurrence of fever, rising inflammatory markers (C-reactive protein (CRP) 298 mg/L), worsening hypoxemia with a decline in the PaO₂/FiO₂ ratio to 168, and radiological progression on repeat high-resolution computed tomography (HRCT), demonstrating increased areas of consolidation and expansion of ground-glass opacities. A milky-appearing bronchoalveolar lavage (BAL) initially yielded negative cultures; however, after 14 days of incubation, it revealed growth of *Nocardia wallacei*, identified by matrix-assisted laser desorption/ionization time-of-flight mass spectrometry (MALDI-TOF MS). A diagnosis of autoimmune PAP was confirmed through the detection of anti-GM-CSF antibodies. The clinical course was complicated by relapse and a left-sided tendon rupture occurring after 10 days of fluoroquinolone therapy. The patient eventually achieved stability through a multidrug regimen followed by long-term secondary prophylaxis with cotrimoxazole for a total of 12 months. This case underscores the diagnostic challenge posed by slow-growing pathogens in patients with underlying rare lung diseases and highlights the necessity of maintaining a high index of suspicion for opportunistic infections when standard treatments fail.

## Introduction

Pulmonary alveolar proteinosis (PAP) is a rare condition characterized by impaired surfactant clearance due to dysfunctional granulocyte-macrophage colony-stimulating factor (GM-CSF) signaling, which disrupts alveolar macrophage-mediated surfactant homeostasis and leads to its accumulation within the alveoli [1]. Autoimmune PAP has an estimated prevalence of approximately 6-7 cases per million individuals and typically affects adults between 30 and 60 years of age, with a male predominance and a strong association with smoking. Clinical presentation is often insidious and characterized by progressive dyspnea, chronic cough, fatigue, and recurrent respiratory infections. Chest radiography (CXR) commonly demonstrates bilateral, symmetric alveolar infiltrates with a perihilar distribution. High-resolution computed tomography (HRCT) classically reveals a “crazy-paving” pattern, defined by ground-glass opacities with superimposed interlobular septal thickening [2]. Diagnosis is supported by bronchoalveolar lavage (BAL) findings of milky fluid containing PAS-positive proteinaceous material and is confirmed by the detection of circulating anti-GM-CSF antibodies. Whole lung lavage remains the standard therapy for moderate to severe disease, while inhaled or subcutaneous GM-CSF replacement therapy may be considered in selected cases [3]. Neutralizing anti-GM-CSF antibodies impair both alveolar macrophage maturation and systemic neutrophil function, leading to a generalized defect in innate immunity rather than a purely localized pulmonary impairment [4].

GM-CSF plays a pivotal role in the terminal differentiation and functional maturation of alveolar macrophages through activation of PU.1-dependent transcription pathways. This signaling cascade is essential for surfactant catabolism, phagolysosomal processing, and intracellular killing of inhaled pathogens. Neutralizing anti-GM-CSF autoantibodies disrupt these mechanisms, leading not only to surfactant accumulation but also to defective macrophage-mediated pathogen clearance. Furthermore, GM-CSF signaling contributes to neutrophil activation and oxidative burst capacity; therefore, its inhibition results in broader impairment of innate immunity. This immunologic dysfunction creates a permissive environment for opportunistic pathogens to establish infection [5]. Among these, Nocardia species are significant. Nocardia spp. are ubiquitous environmental organisms acquired primarily via inhalation and frequently present with nonspecific radiological findings that can mimic other pneumonias [6].

## Case presentation

A 48-year-old male with a significant smoking history of 55 pack-years presented with a history of three hospitalizations over the preceding four years for community-acquired pneumonia. These episodes were characterized by fever, productive cough, and dyspnea, with radiologic evidence of bilateral infiltrates. Each previous event had been managed with empiric intravenous antibiotics, including beta-lactams and macrolides, resulting in symptomatic relief but incomplete radiologic resolution according to available HRCT reports, which consistently described persistent bilateral ground-glass opacities, supporting the hypothesis of longstanding PAP. Notably, the patient was not taking any chronic medications at home, and his remaining medical history was otherwise unremarkable. Regarding his occupational history, he worked in sewage systems, an environment associated with exposure to various environmental pathogens. In 2018, suspicion of PAP was raised by the pulmonology team during a multidisciplinary discussion based on recurrent bilateral infiltrates, incomplete radiologic resolution, and the presence of a crazy-paving pattern on HRCT. At that time, pulmonologists recommended further diagnostic evaluation, including pulmonary function tests and BAL. However, these investigations were not performed. Subsequent healthcare disruptions related to the COVID-19 pandemic beginning in 2020 contributed to additional delays in reassessment and follow-up, ultimately resulting in prolonged diagnostic latency.

On admission, the patient reported high fever, productive cough with purulent sputum, progressive exertional dyspnea, and generalized fatigue. He denied hemoptysis or chest pain. Physical examination revealed tachypnea, with a respiratory rate of 24 breaths per minute, oxygen saturation of 89% on room air, bilateral inspiratory crackles predominantly at the lung bases, and no peripheral edema. Cardiovascular and abdominal examinations were unremarkable. No cutaneous lesions or neurologic deficits were observed.

Laboratory tests on admission revealed a white blood cell count of 21.9 × 10³/µL (neutrophils 15.39 × 10³/µL) and a C-reactive protein level of 251.2 mg/L (Table 1). Additional examinations were performed. Serum lactate dehydrogenase (LDH) was elevated at 412 U/L (normal range 135-225 U/L), consistent with possible ongoing alveolar epithelial injury, and procalcitonin was 1.8 µg/L, supporting the initial suspicion of bacterial pneumonia. Arterial blood gas analysis on room air showed hypoxemic respiratory failure, with a PaO₂ of 58 mmHg and a PaCO₂ of 34 mmHg, corresponding to a PaO₂/FiO₂ ratio of 221. During clinical observation, the PaO₂/FiO₂ ratio further declined to 168, confirming objective deterioration.

**Table 1:** Laboratory tests performed in the emergency department

Test	Obtained Value	Normal Range
White blood cells (WBC)	21.9 ×10³/µL	4-10 ×10³/µL
Neutrophils	15.39 ×10³/µL	2-8 ×10³/µL
Hemoglobin (Hb)	13.7 g/dL	13-18 g/dL
Platelets	209,000 /µL	100,000-395,000 /µL
C-reactive protein (CRP)	251.2 mg/L	0-5 mg/L
Creatinine	0.99 mg/dL	0.7-1.3 mg/dL
Sodium (Na)	137 mEq/L	135-145 mEq/L
Potassium (K)	3.8 mEq/L	3.5-5.0 mEq/L
Alanine aminotransferase (ALT)	22 U/L	7-56 U/L
Total bilirubin	0.7 mg/dL	0.3-1.0 mg/dL

**Figure 1:**
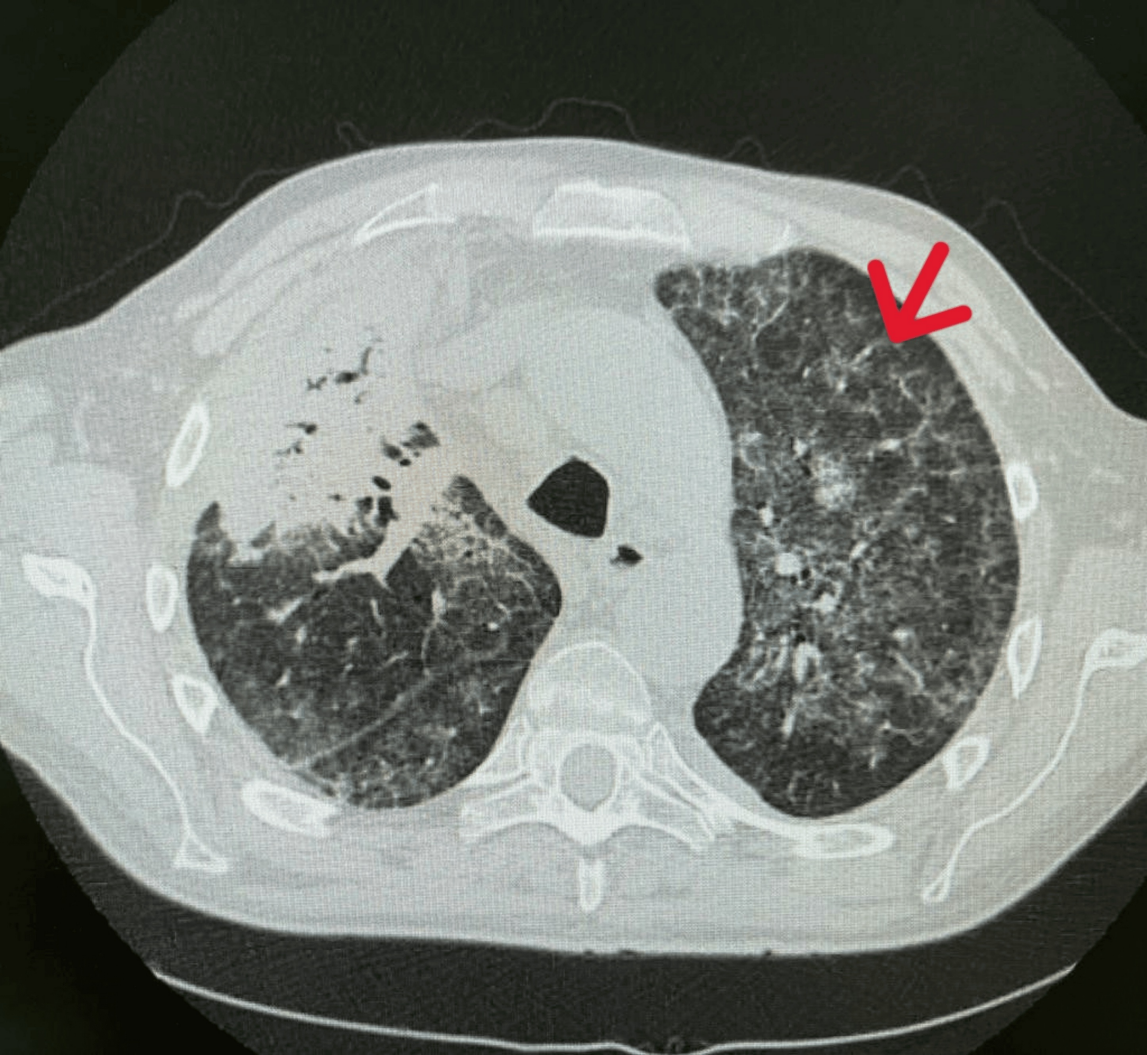
Admission chest high-resolution computed tomography (HRCT). The axial HRCT scan shows diffuse ground-glass opacities associated with interlobular and intralobular septal thickening, creating a "crazy-paving" pattern characteristic of pulmonary alveolar proteinosis. Patchy areas of consolidation are also visible in the dependent lung regions, suggesting superimposed infection.

Initial chest X-ray demonstrated bilateral patchy alveolar infiltrates with perihilar predominance and relative sparing of the lung periphery. Given the nonspecific findings and clinical deterioration, HRCT was subsequently performed for further characterization. Chest HRCT demonstrated extensive parenchymal consolidation in the left lower lobe and right upper lobe, associated with widespread crazy-paving and ground-glass patterns (Figure 1) [7].

Initial therapy included oxygen, empiric broad-spectrum antibiotics (piperacillin/tazobactam and linezolid), and corticosteroids. The treatment plan was established through multidisciplinary consensus.

Intravenous methylprednisolone (40 mg twice daily) was initiated because of severe hypoxemia and radiologic features suggestive of inflammatory interstitial lung disease, following prior pulmonology consultation and before definitive microbiological confirmation. In retrospect, corticosteroid administration (given intravenously at full dosage for 72 hours, followed by gradual tapering over a total of seven days) may have contributed to transient immunosuppression in the context of an unrecognized opportunistic infection.

Linezolid was incorporated into the empiric antimicrobial regimen after consultation with the infectious diseases team to ensure coverage against resistant Gram-positive pathogens, including methicillin-resistant *Staphylococcus aureus *(MRSA), particularly given the patient’s history of recurrent hospitalizations and occupational exposure risk.

BAL fluid appeared milky, and cytological examination revealed abundant foamy macrophages with periodic acid-Schiff (PAS)-positive proteinaceous material, supporting the presumptive diagnosis of PAP [3].

Despite a transient clinical improvement (defined by reduction in fever, decreased oxygen requirement (from 4 L/min nasal cannula to room air), improved PaO₂/FiO₂ ratio (from 168 to 260), decreased cough frequency and sputum production), fever recurred on the 10th day of therapy.

Two sets of blood cultures obtained at emergency department admission and repeated upon transfer to our department remained negative. In the setting of persistent hypoxemia and non-diagnostic initial microbiological investigations, bronchoscopy with BAL was performed following the recommendation of the pulmonology team to evaluate for opportunistic pathogens and to further investigate the suspected diagnosis of PAP.

On the 14th day, the BAL culture became positive for Nocardia wallacei, and the diagnosis was further supported by histopathological findings from transbronchial biopsy (with filamentous branching organisms visualized using modified acid-fast staining). Antimicrobial susceptibility testing demonstrated sensitivity to cotrimoxazole (MIC 0.5 µg/mL) and linezolid (MIC 1 µg/mL), with resistance to amikacin and reduced susceptibility to fluoroquinolones. Antimicrobial escalation was implemented after receipt of these susceptibility results to ensure evidence-based therapy. The diagnosis of autoimmune PAP was confirmed upon detection of positive anti-GM-CSF antibodies [8].

The patient initially received empiric intravenous therapy with piperacillin/tazobactam (4.5 g every six hours) and linezolid (600 mg twice daily) for seven days. Following recurrence of fever (with repeatedly negative blood cultures (negative COVID, FLU, and MRSA swabs)), antimicrobial therapy was escalated to a multidrug regimen consisting of levofloxacin (750 mg daily), linezolid (600 mg twice daily), and meropenem (2 g every eight hours) for 14 days (consulting infectious disease specialist). Given the recognized susceptibility of patients with autoimmune PAP to viral reactivation and the detection of cytomegalovirus (CMV) viremia via quantitative PCR, antiviral therapy with ganciclovir was initiated. Therapy was guided by the presence of viremia in the clinical context rather than by predefined viral load thresholds.

Ten days after initiation of levofloxacin, the patient developed acute left Achilles tendon pain and functional impairment. Musculoskeletal ultrasound of the left Achilles tendon demonstrated a subtotal rupture (with a discontinuous and markedly heterogeneous tendon appearance over approximately 3 cm), confirmed by MRI. Orthopedic consultation confirmed the subcutaneous subtotal rupture; immobilization in slight equinus and non-weight-bearing ambulation with two crutches were prescribed. Surgical repair was indicated but deferred due to comorbidities and the need to first address acute pulmonary issues. Levofloxacin was immediately discontinued.

Eventually, the patient achieved stable clinical and radiological improvement on long-term secondary prophylaxis with cotrimoxazole [9].

## Discussion

This case illustrates several clinically relevant challenges: delayed microbiological confirmation due to the slow-growing nature of Nocardia spp., requiring extended incubation (>14 days); the difficulty in distinguishing infectious progression from inflammatory exacerbation in PAP; and the need for multidrug therapy in the setting of relapse.

Evidence regarding nocardiosis complicating autoimmune PAP is very limited, with only a few cases reported. The limited number of reported cases further emphasizes the need for detailed case documentation to improve understanding of infectious complications in autoimmune PAP.

Managing nocardiosis in PAP patients is challenging due to the slow growth of the bacteria, which often requires more than 14 days to appear in culture [6]. Standard therapies such as cotrimoxazole and linezolid remain effective against over 95% of Nocardia isolates [6]. In the context of PAP, the neutralizing effect of anti-GM-CSF antibodies not only leads to surfactant accumulation but also severely impairs neutrophil and macrophage bactericidal capacity, increasing the risk of recurrence [2,8]. Long-term antibiotic treatment (6-12 months) and secondary prophylaxis are generally necessary to ensure eradication, especially when whole lung lavage (WLL) is not immediately performed [9].

In this case, the total planned duration of antimicrobial therapy was 12 months, considering the patient’s underlying immune dysfunction. Secondary prophylaxis with trimethoprim-sulfamethoxazole was continued thereafter, with periodic clinical and radiologic monitoring.

The internist plays a crucial role in managing such clinical complexity, ensuring timely diagnosis and referral to specialized centers for advanced immunologic testing and therapeutic interventions such as whole lung lavage.

## Conclusions

In patients with suspected or confirmed autoimmune PAP who deteriorate despite empiric antibiotic therapy, prolonged microbiological incubation and repeated bronchoscopy should be considered to exclude slow-growing opportunistic pathogens. The presence of a crazy-paving pattern on HRCT, particularly in patients who deteriorate despite empiric therapy, should prompt investigation for opportunistic infections, including nocardiosis. Early diagnosis of the specific PAP form is essential for timely referral to specialized centers and appropriate therapeutic management.

In this case, diagnostic timing was determined primarily by the prolonged incubation required for Nocardia growth rather than by radiologic pattern recognition alone. Although the presence of a crazy-paving pattern raised suspicion for underlying autoimmune PAP, microbiological confirmation was delayed due to the slow-growing nature of the pathogen. Objective indicators of clinical deterioration, including a declining PaO₂/FiO₂ ratio, persistently elevated inflammatory markers, and radiologic progression, were pivotal in prompting repeated diagnostic reassessment and therapeutic escalation.

This case underscores the need for extended microbiological surveillance in patients with autoimmune PAP who fail to respond to empiric therapy, as well as the importance of systematic monitoring for treatment-related adverse events during prolonged antimicrobial regimens.
